# Modulatory effect of resveratrol and melatonin on natural killer cell activity and adrenomedullin in diabetic rats

**DOI:** 10.3906/sag-2104-380

**Published:** 2021-06-24

**Authors:** Fatih ÖZÇELİK, Fatih HACIMUSTAFAOĞLU, Alpaslan TANOĞLU

**Affiliations:** 1 Department of Medical Biochemistry, Sultan 2. Abdulhamid Han Training and Research Hospital, University of Health Sciences Turkey, İstanbul, Turkey; 2 Department of Medical Biochemistry, Hamidiye Faculty of Medicine, University of Health Sciences Turkey, İstanbul, Turkey; 3 Department of Gastroenterology, Sancaktepe Training and Research Hospital, University of Health Sciences Turkey, İstanbul, Turkey

**Keywords:** Diabetes mellitus, melatonin, resveratrol, natural killer cell activity, mid-regional proadrenomedullin

## Abstract

**Background/aim:**

Epidemiological evidence suggests that diabetes poses a high risk for many chronic diseases, especially cardiovascular diseases, and cancer by stimulating many inflammatory and immunological pathogenic mediators and affecting natural killer (NK)-cell activity. In this study, the effects of melatonin and resveratrol on IL-6, TNF-alpha, oxidant/antioxidant capacity, NK-cell activity, and mid-regional proadrenomedullin (MR-proADM) levels of diabetic rats were investigated.

**Materials and methods:**

In the study, 28 Sprague Dawley rats were randomly divided into the control group (group I) and 3 streptozotocin-induced diabetes mellitus (DM) groups (group II, III, and IV), each group consisting of 7 rats. Five mg/kg/day melatonin to group III and 5 mg/kg/day resveratrol (intraperitoneal) to group IV was given. At the end of 3 weeks, NK-cell activity, total antioxidant/oxidant capacity, MR-proADM, IL-6, and TNF-alpha levels were measured in intracardiac blood taken under anesthesia.

**Results:**

NK-cell activity of group II was found lower than group I, group III, and group IV (7.4 ± 2.0 vs. 22.5 ± 11.9, 30.6 ± 22.5 and 20.4 ± 9.1 pg/mL; p = 0.0018, respectively). The difference was more prominent in diabetic rats receiving melatonin (p < 0.01). TNF-alpha levels of group II were higher than the group I (p < 0.05). The MR-proADM levels of group II were found to be lower than the group I and group III (6.4 ± 3.6 vs. 14.4 ± 3.2 and 14.0 ± 4.2 ng/L; p < 0.05, respectively). In addition, NK-cell activity was moderately correlated with MR-proADM (r = 0.5618, p = 0.0019).

**Conclusion:**

Resveratrol and, more effectively, melatonin modulate by reversing the adverse effects of diabetes on NK-cell activity, which has a protective function in inflammatory and immunological processes. In this modulation, melatonin also acts through adrenomedullin.

## 1. Introduction

Diabetes mellitus (DM), whose incidence is constantly increasing globally, is a chronic and serious metabolic disease causing the deadliest health problems worldwide. Epidemiological evidence suggests that DM poses a high risk for many types of diseases, including cancer, primarily cardiovascular. Inflammatory, immunological, and endocrinological factors that are effective in this deterioration transforming process of DM appear to be associated with the incidence or mortality of the diseases [1,2]. Every day, the structure of thousands of cells in our body deteriorates due to various microorganisms, harmful substances, rays, and chronic diseases such as DM. Natural killer (NK) cells, which are a kind of lymphocyte cell and have a wide variety of biological activities, can correct inflammation in tissues invaded by pathogens. They can recognize disrupted cells that tend to differentiate into cancer from the changed antigenic structure and destroy them. In addition, they can also eliminate immune dysfunction. For this reason, NK-cell count is very important in the body’s fight against diseases. However, recently it has been realized that NK-cell activity is more important than NK-cell count because it has been observed that although the NK-cell count is high, they cannot fulfill their protective functions against cancer [3,4]. In addition to many chronic adverse effects of DM, it was found to impair glucose transporter (GLUT) protein expression and decrease NK-cell activity associated with it. This suggests that diabetes has an important role in the pathogenesis of many chronic diseases. In addition, today, abnormalities in oxidant and antioxidant systems, growth factors, adrenomedullin (ADM), inflammatory cytokines (interleukin-1beta (IL-1β)), various interleukins (IL-6, IL-15, IL-17, and IL-18), tumor necrosis factor-alpha (TNF-α), and chemokines are considered to contribute to the pathogenesis of DM-related disorders, inflammatory diseases, and cancers by activating various transcription factors [4–7]. Therefore, the determination of direct or indirect relationships with NK-cell activity of agents such as melatonin and resveratrol [8,9], which are known to be associated with the above biological mediators, may lead to finding preventive treatment options that can prevent or reduce the degradation of normal cells.

In this study, a completely insulin-free in vivo diabetic environment was created using streptozotocin to induce the adverse effects on NK-cell activity by disrupting the expression of insulin-dependent GLUTs in rats’ NK-cells. Subsequently, the effects of resveratrol and melatonin on NK-cell activity were investigated. In addition, the changes in total antioxidant/oxidant status, mid-regional proADM (MR-proADM is a more stable part of proADM than ADM and is more suitable for measurement), IL-6 and TNF-α levels, which we think may be associated with NK-cell activity, were examined.

## 2. Material and methods

### 2.1. Experimental protocol

Approval for the study was obtained from the University of Health Sciences Animal Experiments Local Ethics Committee (protocol number: 46418926-605.02, 2019-08/02). The research was planned as an experimental study with analytical prospective study type. This study was carried out between May and June 2020. A total of 28 Sprague Dawley, male, adult rats, 16–20 weeks old and weighing 250–350 g, were used. Standard care and nutrition were given to the rats. Rats were kept in laboratory conditions at an ambient temperature of 20–22 °C and in a circadian rhythm of 12 h night and 12 h day. Rats were randomized into four groups (group I, II, III and IV) containing 7 rats in each. All rats were fasted for 12 h before the intracardiac blood collection. Then their body weights were recorded. A single dose of 60 mg/kg streptozotocin dissolved in (0.1 M) sodium-citrate buffer (pH 4.5) was administered intraperitoneally (IP) to groups II, III, and IV for the development of DM. Then, 48 h after the administration of streptozotocin, glucose levels were measured with a glucometer from a drop of blood taken from the dorsal veins of the hind feet of rats. Rats with blood glucose levels above 200 mg/dL were considered as diabetic [10].

### 2.2. Rat groups

#### Group I (control group)

To eliminate the effects of the sodium-citrate buffer, this non-diabetic group was given the same amount of IP sodium-citrate buffer as used to dissolve streptozotocin. In addition, 25% ethanol-saline solution used as the solvent of melatonin and resveratrol was given as IP in the same amount as the other groups every day between 18:00 and 19:00 for 3 weeks. The aim here was to eliminate the possible effects of ethanol-saline solution in which melatonin and resveratrol are dissolved.

#### Group II (DM group)

As was done in group I, 25% ethanol-saline solution was given to this diabetic control group as IP every day between 18:00 and 19:00 for 3 weeks.

#### Group III (DM + Melatonin group)

In this diabetic group, melatonin prepared by dissolving in 25% ethanol-saline solution was administered at a dose of 5 mg/kg/day for 3 weeks between 18:00 and 19:00 [11]. The rats were taken to a dark environment after the injection [12]. To better observe the melatonin-related effects, the rats in groups I, II and IV, which were not given melatonin, were also taken to the dark-light period with a similar application.

#### Group IV (DM + Resveratrol group)

In this diabetic group, resveratrol prepared by dissolving in 25% ethanol-saline solution was injected IP at a dose of 5 mg/kg/day for 3 weeks between 18:00 and 19:00 [13].

Streptozotocin (≥ 75% α-anomer basis, ≥ 98% HPLC), melatonin (≥ 98% TLC) and resveratrol (≥ 99% HPLC) were obtained from Sigma-Aldrich (Saint Louis, USA).

### 2.3. Surgical procedures

The same general anesthesia was applied to all rats. Anesthesia was provided by IP administration of a combination of ketamine 100 mg/kg and xylazine 10 mg/kg. Anesthesia was applied 30 min before the surgical procedure. When necessary, 1/3 of the initial dose was repeated as a maintenance dose. All rats were sacrificed by cervical dislocation after intracardiac blood collection at the end of the third week under anesthesia. Some of the blood taken from the rats was put into NK activity tubes specially prepared for the NK-cell activity test. NK activity tubes were removed from the refrigerator just before blood collection and the procedure was performed within 5 min. The plasma obtained from the blood in these NK activity tubes was stored at −80 ^o^C until measurement day (maximum 3 months). Blood from rats to serum tubes was centrifuged at 3500 RPM for 10 min. Sera separated by centrifugation were stored at −80 ^o^C until the measurement day. Total antioxidant status (TAS), total oxidant status (TOS), MR-proADM, IL-6, and TNF-α levels were measured in these sera.

### 2.4. Analysis of biochemical parameters

#### NK-Cell activity levels

NK-cell activity test enzyme-linked immunosorbent assay (ELISA) kit (ATGen, Seongnam-si, South Korea) was used for NK-cell activity measurement. The sensitivity of the test kit was 40 pg/ml and the measuring range was 40–2000 pg/mL.

Special NK activity tubes (ATGen, Seongnam-si, South Korea) used for NK-cell activity tests are vacuum blood collection tubes containing Promoca and heparin as anticoagulant. Immediately after blood was drawn into NK activity tubes, the contents of the tube were gently inverted and mixed. 15 min after blood collection, NK activity tubes were transferred to the incubator. Attention was paid to not cool the blood collected in NK activity tubes before incubation. NK activity tubes containing blood were incubated at 37 ^o^C for 20–24 h to obtain the appropriate response from NK-cells in the blood. At the end of the incubation, the clear or yellowish clear plasma which remained on top was carefully transferred into microcentrifuge tubes. This plasma was stored in a refrigerator at +2 °C to 8 °C for 1 day or at –20 °C for 3 months until the study was performed. Plasma samples frozen before the study should be thawed at room temperature and then centrifuged at 11,500 g for 1 min at room temperature.

NK activity tubes contained a stimulating stabilized immunomodulatory cytokine called Promoca. During the incubation process, cytokine-stimulated NK-cells were induced to secrete interferon-gamma (IFN-) into plasma. IFN-levels were measured as absorbance at 450 nm in ELISA device (BioTek Epoch 2 Microplate Spectrophotometer and ELx50 Microplate Strip Washer, USA).

#### Rat IL-6 and TNF-α levels

Serum IL-6 and TNF-α levels of the blood samples were analyzed using the double antibody sandwich-ELISA method (Elabscience Biotechnology Co. Ltd, Wuhan, China). The sensitivity of the IL-6 assay is 7.5 pg/mL, the measuring range is 12.5–800 pg/mL, and the CV is < 10%. The sensitivity of the TNF-α assay is 46.9 pg/mL, the measuring range is 78.1–5000 pg/mL, and the CV is < 10%.

#### Rat MR-proADM levels

Rat serum MR-proADM levels were analyzed using double antibody sandwich-ELISA method kits (Bioassay Technology Laboratory, Shanghai, China). The sensitivity of MR-proADM assay was 0.58 ng/L, measuring range 1–380 ng/L, and intraassay and interassay CV were < 8% and < 10%, respectively.

#### Rat total oxidant/antioxidant status

Rat TOS and TAS levels were analyzed using double antibody sandwich-ELISA method kits (Bioassay Technology Laboratory, Shanghai, China). The sensitivity of both TOS and TAS test kits was specified as 0.013 U/mL, measurement range 0.2–60 U/mL, and intraassay and interassay CVs < 8% and < 10%.

### 2.5. Statistical analysis

IBM SPSS Statistics v: 20.0 ( IBM Corp., Chicago, USA) Software was used for statistical analysis of all data. In comparison of statistical data with normal distribution and containing more than two independent groups, one-way ANOVA with Post-Hoc test was used. If the data did not have a normal distribution, the Kruskal–Wallis with Post-Hoc test was used. Pearson correlation analysis was used for correlational studies of data with parametric distribution, and Spearman correlation analysis was used for nonparametric data.

## 3. Results

### 3.1. Characteristics of rats used in the experimental study

In the study, there was no statistical difference between the mean ages and weights of the rats included in group I, II, III, and IV (age: 17.9 ± 1.3, 18.1 ± 1.2, 17.7 ± 1.7 and 17.9 ± 0.9 weeks, p = 0.9077; 298 ± 20, 294 ± 19, 289 ± 24 and 292 ± 24 g, p = 0.7219, respectively) (Table 1).

### 3.2. Comparisons between groups

**Figure 1:**
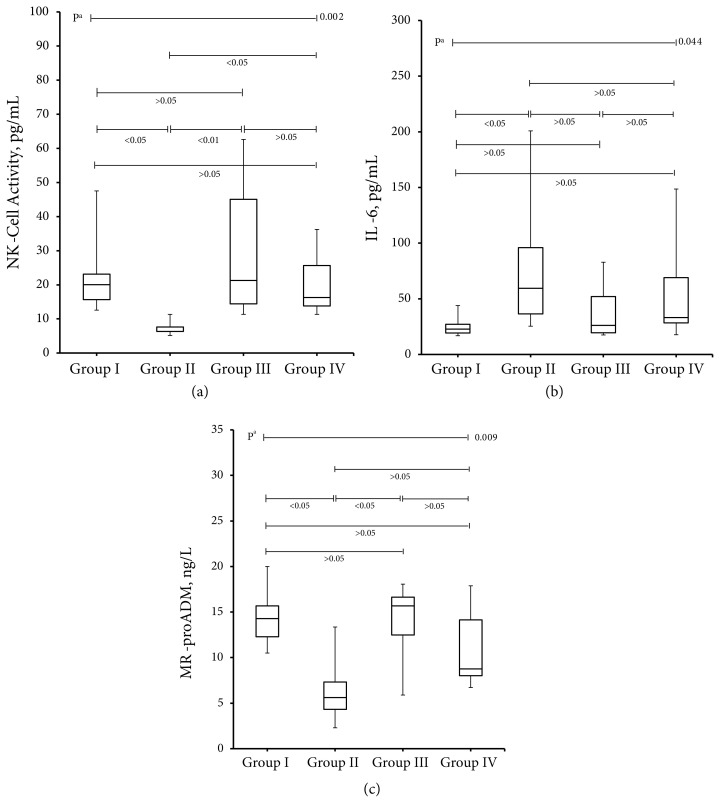
Box plots graph comparing NK-cell activities, IL-6 levels and MR-proADM levels in control group (group I), diabetic group (group II), DM + melatonin (group III) and DM + resveratrol group (group IV). The NK-cell activities, IL-6 levels, and MR-proADM levels were compared with the Kruskal-Wallis test (nonparametric ANOVA) due to the number of samples in the groups. A) While NK-cell activity was lower in the diabetic group compared to the control group, NK-cell activity increased again in the diabetic groups receiving melatonin and resveratrol. B) IL-6 levels appear to be higher in the diabetic group compared to the control group. However, the values in the diabetic groups administered melatonin and resveratrol are not different from the control group. C) MR-proADM levels are lower in the diabetic group compared to the control group. However, there is no difference in the diabetic groups receiving melatonin and resveratrol compared to the control group. Compared to the diabetic group, rats receiving melatonin had higher MR-proADM levels.

When the experimental groups were evaluated, the NK-cell activity of group II was found to be lower than Group-I (7.4 ± 2.0 vs. 22.5 ± 11.9 U/mL, respectively; p < 0.05) (Table 1). The NK-cell activity was higher in diabetic rats in group III and group IV compared to group II (30.6 ± 22.5 and 20.4 ± 9.1 vs. 7.4 ± 2.0 U/mL; p < 0.05, respectively). The difference was more prominent in diabetic rats receiving melatonin (p < 0.01) (Figure 1A). The NK-cell activity of diabetic rats in group III and group IV was not statistically different from group I (p > 0.05). Similarly, there was no difference between NK-cell activities of diabetic rats in group III and group IV (30.6 ± 22.5 vs. 20.4 ± 9.1 U/mL; p > 0.05, respectively).

When the experimental groups were evaluated in terms of IL-6, levels in group II were much higher compared to the group I (78.6 ± 62.0 vs. 25.2 ± 9.3 pg/mL; p < 0.05, respectively) (Table 1). Although IL-6 levels of diabetic rats in group III and group IV were lower compared to group II, the difference was not statistically significant (38.5 ± 27.6 and 56.3 ± 45.6 vs. 78.6 ± 62.0 pg/mL; p > 0.05, respectively) (Figure 1B). In addition, it was noteworthy that there was no difference among IL-6 levels of rats in group I, III, and IV (25.2 ± 9.3 vs. 38.5 ± 27.6 and 56.3 ± 45.6 pg/mL; p > 0.05, respectively).

The MR-proADM levels in group II were lower compared to group I and group III (6.4 ± 3.6 vs. 14.4 ± 3.2 and 14.0 ± 4.2 ng/L; p < 0.05, respectively), while the MR-proADM levels of group IV were not different (6.4 ± 3 vs. 11.1 ± 4.3 ng/L; p > 0.05, respectively) (Table 1 and Figure 1C). However, MR-proADM levels of diabetic rats in group III and group IV were not statistically different compared to group I (14.0 ± 4.2 and 11.1 ± 4.3 vs. 14.4 ± 3.2 ng/L; p > 0.05, respectively). Similarly, there was no difference between MR-proADM levels of group III and IV diabetic rats (14.0 ± 4.2.6 vs. 11.1 ± 4.3 ng/L; p > 0.05, respectively).

**Figure 2:**
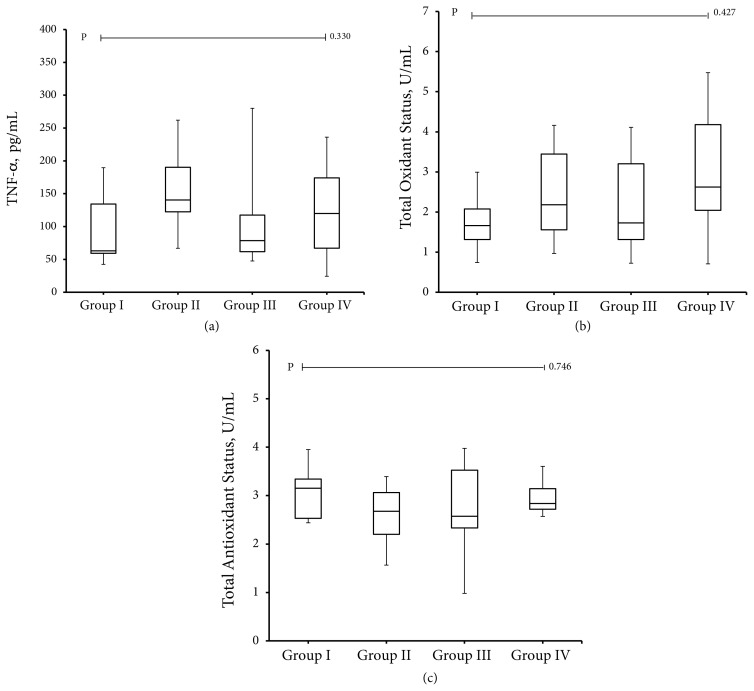
Box plots graph comparing control group (group I), diabetic group (group II), DM + melatonin (group III), and DM + resveratrol groups (group IV) in terms of TNF-α, TAS and TOS levels. There is no difference between the groups in terms of these parameters.

All groups were not different in terms of TNF-α, TAS, TOS (p > 0.05) (Table 1) (Figures 2A-C). However, we think that this situation should be confirmed with a larger population.

### 3.3. Correlational studies

**Figure 3:**
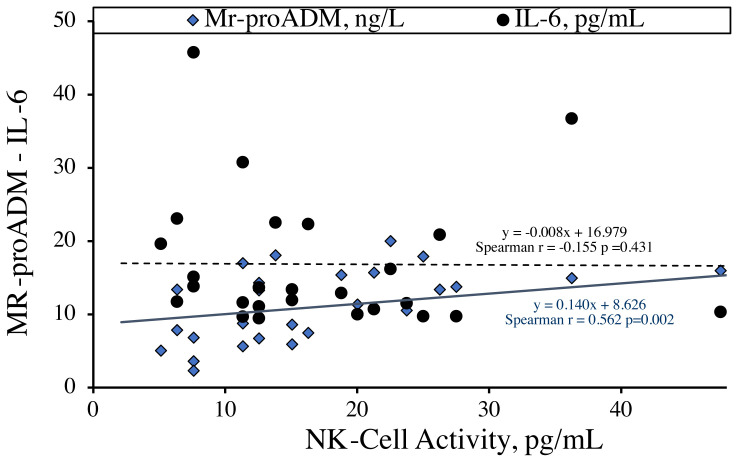
Correlation plot showing the relationship between NK-cell activity, MR-proADM, and IL-6 levels. The graph shows the direct proportional correlation of NK cell activity with MR-proADM.

According to the correlation analysis results, NK-cell activity showed a positive correlation with MR-proADM (Spearman rs = 0.562, p = 0.0019) (Table 2), but the correlation with TAS and TNF-α became statistically insignificant (Spearman r = 0.208, p = 0.2873, Spearman r = −0.318; p = 0.0994, respectively) (Figure 3). Also, there was no significant correlation between NK-cell activity and IL-6 (Spearman r = −0.1550, p = 0.4311). Moreover, the significant correlation between IL-6 and TOS was found to decrease to r = 0.389 (p = 0.0407).

## 4. Discussion

Diabetes is a multifaceted disorder that occurs in glucose use and insulin-related metabolic processes. Genetic predisposition, lifestyle, diet, limited physical activity, obesity, aging, and immunological disorders can also trigger DM. All these risk factors can reveal common pathogenic mediators of diabetes by stimulating the inflammatory and immunological pathways. Metabolic models created by diabetes offer an effective area of investigation in understanding the possible mechanisms that direct inflammatory and immunological pathways. In this way, it is possible to prevent or control diabetes and diabetes-related pathologies through the resulting responses [2,14].

NK-cells, which make up about 5%–19% of blood lymphocytes, protect the body against infection, abnormal inflammatory response, and cancer by activation through various cell surface receptors. They can secrete cytokines through the CD56/CD16 receptor and destroy infected, disrupted, or cancerous target cells with cytotoxic granule exocytosis and antibody dependent cellular toxicity. They are also effective in shaping the acquired immune response [15,16]. In addition, low NK-cell activity is thought to be associated with type 1 DM pathogenesis [17]. Some agents such as vitamins, polysaccharides, and lectins obtained from plant extracts, which have stimulating properties for NK-cells, are considered as an option for treatment to benefit from these functions of NK-cells [18]. If these options can stimulate NK-cell activity at an adequate level, the development of many complications of diabetes, which tend to increase especially with aging, will be preventable.

It has been known that peripheral blood lymphocytes are particularly sensitive to changes in blood glucose levels. In addition, lymphocytes are more durable than granulocytes and can be obtained easily [19]. Therefore, the use of lymphocytes (NK-cells) in the investigation of the chronic effects of diabetes is very appropriate. In our study, we used diabetic rats to better observe the changes in NK-cell activity. Thought to modulate the immune system associated with NK-cell activity in recent years, resveratrol is a stilbene compound (phytoalexins) often synthesized by plants in response to stressful stimuli [20]. It was reported that this compound has an anti-hyperglycemic effect by stimulating the intracellular transport of glucose in diabetic animals [21]. In our experimental rat study, the NK-cell activity was much lower in the diabetic rat group compared to the control group, while the high level in diabetic rats receiving resveratrol was consistent with the above information indicating its efficacy. This finding is evidence of the effect of resveratrol on NK-cell activity. The reason for the low NK-cell activity found in diabetic rats was attributed to the decrease in glucose transport to NK-cells in the absence of insulin. Hence, NK-activity will also slow down as it will lack energy in the absence of insulin [5]. In this current study, the intake of resveratrol may have been eliminated the negative effect caused by the lack of insulin so that NK-cell activity was restored. This hypothesis is in line with the information reported by Vallianou et al. [21], in 2013, that resveratrol increases the expression of the glucose transporter GLUT4 by showing an insulin-like effect. A similar study found that resveratrol improved insulin resistance and glucose homeostasis in obese men with Caucasian metabolic syndrome [22]. However, all these results are not sufficient to explain the precise mechanism of resveratrol in NK-cell activity and there is still a need for detailed molecular research.

In a study by Kimura and Okuda investigating metastatic lung carcinoma, they found that resveratrol significantly reduced tumor volume, weight, and lung metastasis only at high doses (2.5 and 10 mg/kg) without affecting the number of CD4+ (helper), CD8+ (cytotoxic), and NK-cells in the spleen [23]. Although this finding seems to contradict our results, they differ from us because they did not evaluate NK-cell activity and, they did not use diabetic subjects. In another study, whose results we think may be related to NK-cell activity, resveratrol has been reported to modulate immune function depending on the dose. The same study also found that low doses of resveratrol stimulate the immune system, while high doses suppress the immune system [24]. In this current study, resveratrol provided a significant increase in NK-cell activity. This situation showed that the dose (5 mg/kg/day) used in rats could be sufficient to stimulate the immune system.

Insulin resistance, insulin-like growth factors, hyperglycemia, hyperlipidemia and inflammatory cytokines were associated with the risk of cancer developing [25]. It is considered that the most likely mechanism that could explain the progression to cancer is a decrease in NK-cell activity and an abnormal inflammatory response due to an imbalance in insulin. In our study, the fact that NK-cell activity and MR-proADM, which has anti-inflammatory properties, were significantly lower and IL-6, a proinflammatory cytokine, was higher in diabetic rats supported this view. In addition, the increase in NK-cell activity and MR-proADM in rats receiving melatonin and resveratrol, despite they are diabetic, unlike IL-6, were promising results in the transformation of pathological processes. Apparently, NK-cell activity in rats was also effective, possibly by accelerating antiinflammatory processes or by suppressing proinflammatory cytokines. In addition, the moderate correlation detected between NK-cell activity and MD-proADM is an important proof of this hypothesis.

Melatonin, a neurohormone synthesized by the pineal gland from tryptophan, is a modulator in immune cell production and functions, including NK-cells and lymphocyte subgroups, as well as providing antioxidant protection by scavenging free radicals. Although the exact mechanism is unknown, it has been reported that melatonin administration stimulates the secretion of cytokines such as IFN-γ, IL-6, IL-2, and TNF-α [26,27] and is associated with type 1 DM [28]. The determination in our study that diabetic rats receiving melatonin have higher NK-cell activity provides useful data to overcome the above information gap. The likely reason for the recovery of this NK-cell activity is that the reduced NK-cell activity due to lack of insulin is substantially recovered via melatonin. This situation was attributed to the melatonin receptor-mediated melatonin-insulin antagonist effect, a member of the G protein coupled receptor (GPCR) family [29,30].

Previously it was reported that IL-6, especially TNF-α, may have an important role in the etiology of diabetes and insulin resistance, and IL-6 levels may increase 2–3 times [31,32]. Contrary to this information, it has been reported that IL-6, a pleiotropic cytokine, does not cause insulin resistance and may even be beneficial for diabetes in some cases [33]. In our study, while the levels of TNF-α were not different in all groups, only the diabetic rat group had high IL-6 levels. The possible reason for this elevation may be the chronic inflammatory effect of diabetes, which develops due to the absence of insulin and is accompanied by low NK-cell activity. Moreover, the high IL-6 level in the serum of the patients as well as the deterioration in NK-cell function in systemic juvenile idiopathic arthritis, which is a chronic inflammatory and autoimmune disorder, supports our view [34]. This finding significantly overlaps with our results.

Another important result of our study was the comparison of melatonin and resveratrol in terms of their effects on NK-cell activity. In this context, there was a much greater increase in NK-cell activity in diabetic rats given melatonin compared to resveratrol. The possible mechanism underlying this has been attributed to melatonin being a powerful stimulus of the antioxidant systems and the ability of melatonin receptors to interact with many different proteins [35,36]. However, the fact that there was no difference between the groups in terms of TAS and TOS in our study suggested that approaches in which more specific components of oxidative damage studies may be needed. Some researchers reported that there may be still unknown oxidants/antioxidants and those different types of oxidants/antioxidants in the same system can interact with each other, confirming this idea [37]. Therefore, it does not seem possible to precisely explain the relationship between the NK-cell activity and oxidative/antioxidative system, which has highly complex molecular mechanisms beyond what is known, based on only TAS and TOS.

Another interesting finding of this study was the determination of serum MR-proADM levels parallel to NK-cell activity. Compared to the control group, both NK-cell activity and MR-proADM levels were lower in diabetic rats, while levels in diabetic rats receiving melatonin were close to the control group. In the literature review we conducted to explain this interesting relationship, it was understood that ADM has very complex effects on various organs and tissues [38]. It increases intracellular Ca^++^ independent from cAMP. In this way, endothelial nitric oxide synthase is activated and vasodilation occurs with nitric oxide release [39]. Additionally, ADM modulates protein kinase G activation (nitric oxide/cGMP/protein kinase G signaling pathway) by increasing the level of guanosine 3′,5′-cyclic monophosphate (cGMP) via nitric oxide [40]. Besides its these complex metabolic functions, ADM was found to be closely related to members of the inflammatory process [41]. ADM also provides antiinflammatory effect by suppressing the release of TNF-α and IL-1β through IL-10, while stimulating proinflammatory cytokine release such as IL-6 and IL-10 [42]. Administration of ADM to transgenic mice restored early insulin resistance in adipose tissue by inhibiting adipocyte major histocompatibility complex class II (MHC II) antigen presentation and CD4 (+) T cell activation [43]. Therefore, it is understood that ADM has a regulatory effect on proinflammatory and antiinflammatory cytokines, members of oxidant/antioxidant system and mediators related to insulin resistance. All the above information explains why while MR-proADM levels were found to be higher in diabetic rats given melatonin compared to diabetic rats, IL-6 levels decreased to the levels of the control group. However, this view needs confirmation.

The results obtained in the study belong to animal experiments. This experimental study is not fully applicable to humans. In conclusion, resveratrol and, more effectively, melatonin modulate by reversing the adverse effects of diabetes on NK-cell activity, which has a protective function in inflammatory and immunological processes. In this modulation, melatonin also acts through adrenomedullin.

## Figures and Tables

**Table 1:** Comparison of the data according to NK-cell activity, IL-6, MR-proADM, TNF-α, total antioxidant status, and total oxidant status.

	Group I	Group II	Group III	Group IV	p
n	7	7	7	7	^-^
Age, weeks	18.0(16.0–20.0)	18.0(17.0–20.0)	17.0(16.0–20.0)	18.0(17.0–19.0)	0.91^a^
Weight, g	300(274–332)	292(271–320)	281(268–339)	286(270–342)	0.72^a^
NK-cell activity, pg/mL*	20.0(12.6–47.5)	7.6(5.1–11.3)	21.3(11.3–62.6)	16.3(11.3–36.2)	0.01^a^
*p from post-test*	<0.05, >0.05, >0.05, <0.01, <0.05, >0.05				
IL-6, pg/mL*	22.8(16.8–44.0)	59.4(25.3–200.8)	26.1(17.5–82.8)	33.1(17.7–148.6)	0.04^a^
*p from post-test*	<0.05, >0.05, >0.05, >0.05, >0.05, >0.05				
MR-proADM, ng/L*	14.3(10.5–20.0)	5.6(2.3–13.4)	15.7(5.9–18.1)	8.8(6.7–17.9)	0.01^a^
*p from post-test*	<0.05, >0.05, >0.05, <0.05, >0.05, >0.05				
TNF-α, pg/mL	63(42–190)	141(67–262)	79(48–80)	120(24–236)	0.33^a^
TAS, U/mL	3.15(2.44–3.95)	2.68(1.56–3.39)	2.57(0.98–3.98)	2.84(2.57–3.61)	0.75^a^
TOS, U/mL	1.66(0.74–2.99)	2.18(0.97–4.16)	1.73(0.72–4.11)	2.62(0.71–5.47)	0.43^a^

a Kruskal–Wallis test,

* If p value obtained by ANOVA is <0.05, p values of between groups (respectively, groups I–II, groups I–III, groups I–IV, groups II–III, groups II–IV and groups III–IV) are compared with post-test and nonparametric data are given as median (min–max).

Group I: Control group, Group II: Diabetes Mellitus (DM) group, Group III: DM+ Melatonin, Group IV: DM+Resveratrol group, g: Gram, NK: Natural killer, IL: Interleukin, MR-proADM: Midregional-proadrenomedullin, TNF-α: Tumor necrosis factor-alpha, TAS: Total antioxidant status, TOS: Total oxidant status.

**Table 2:** Correlation results of data for all rat groups.

n:28	Spearman’s correlation coefficient (rs)					
	A:	B:	C:	D:	E:	F:
A:NK-cell activity	1.000					
B:IL-6	−0.155	1.000				
C:MR-proADM	0.562**	−0.383*	1.000			
D:TNF-α	−0.318	0.336	−0.360	1.000		
E:TAS	0.208	−0.150	0.061	−0.163	1.000	
F:TOS	0.068	0.389*	−0.2266	−0.068	0.054	1.000

* The P value is < 0.05.

** The P value is < 0.01.

The p-value of insignificant correlations was not given. Spearman’s correlation results are evaluated according to the nonparametric data.
